# Prevalence of inflammatory bowel disease across states: estimates based on claims database

**DOI:** 10.1093/ibd/izag076

**Published:** 2026-05-18

**Authors:** Hansol Kang, Kate L Karlin, Colleen M Brensinger, Orna G Ehrlich, Michael D Kappelman, Alan Moss, James D Lewis

**Affiliations:** Division of Gastroenterology and Hepatology, Perelman School of Medicine, University of Pennsylvania, Philadelphia, PA 19104, United States; Division of Gastroenterology and Hepatology, Perelman School of Medicine, University of Pennsylvania, Philadelphia, PA 19104, United States; Center for Clinical Epidemiology and Biostatistics, Perelman School of Medicine, University of Pennsylvania, Philadelphia, PA 19104, United States; Crohn’s & Colitis Foundation, New York, NY 10017, United States; Department of Pediatrics, Division of Pediatric Gastroenterology, University of North Carolina Chapel Hill, Chapel Hill, NC 27599, United States; Crohn’s & Colitis Foundation, New York, NY 10017, United States; Division of Gastroenterology and Hepatology, Perelman School of Medicine, University of Pennsylvania, Philadelphia, PA 19104, United States; Center for Clinical Epidemiology and Biostatistics, Perelman School of Medicine, University of Pennsylvania, Philadelphia, PA 19104, United States

**Keywords:** inflammatory bowel disease, prevalence, United States, state-level, claims

## Abstract

Data derived from our previously published pooled administrative claims database analysis were applied to estimate race-weighted state-specific prevalences of inflammatory bowel diseases in the United States to support public health, clinical, and research initiatives at the state and regional level.

## Introduction

The United States has among the highest incidence and prevalence of inflammatory bowel diseases (IBDs), which include ulcerative colitis (UC) and Crohn disease (CD).^1^^,^^2^ Prior estimates to quantify the incidence and prevalence of IBD in the United States have been challenging due to the lack of a united health system or comprehensive population-based tracking registries for IBD.^3^ As a result, data are scarce regarding the geographic distribution of IBD in the United States^4^ To address this knowledge deficit, we previously pooled administrative claims datasets to estimate the incidence and prevalence of IBD within the United States^5^ The study found the prevalence to be 721 per 100 000 population, extrapolating to an estimate of 2.39 million Americans, among whom 1.25 million Americans are estimated to have UC and 1.01 million Americans are estimated to have CD.^5^ The study also evaluated geographic and racial distributions of IBD and found that IBD prevalence was highest in the Northeast and lowest in the West as well as highest in White Americans in comparison to Black, Asian, and Hispanic Americans.^5^

Given the diversity of the United States population, there is a further need to understand how the geographic distribution of IBD varies state by state, which remains poorly understood. State-level IBD estimates are important for nationwide high-quality IBD care because health care planning, allocation of specialty care resources, and public health interventions often occur at the state level. Understanding the geographic distribution of IBD on a more granular level may also help identify regions with higher disease burden, inform workforce and health care infrastructure planning, and guide future research into social determinants of health contributing to variations in disease prevalence and management.^6^

In 2026, Upadhyay et al. published state-level IBD prevalences based on estimates generated from an open-source IQVIA patient-centric medical claims database.^7^ However, this study estimated the United States IBD prevalence to be 520 020 Americans in contrast to the 2.39 million in our prior study and also used inclusion criteria to identify IBD patients that were different from those used in our study.^5^^,^^7^ Given the discrepancies and need for state-level data, we aimed to estimate the geographic prevalence of IBD by state. With this objective in mind, we applied the data derived from the pooled administrative claims database analysis. These data were further augmented to obtain race-weighted IBD prevalence estimates by state to account for differences in racial demographics, as the percentage of White Americans has been reported to vary from as high as 94.2% in Maine to as low as 25.2% in Hawaii.^8^

## Methods

### Data sources

The national estimates of IBD prevalence were derived from 4 administrative claims datasets as previously described.^5^ The datasets include Medicare, Medicaid, and 2 commercial health insurance datasets including Optum’s deidentified Clinformatics Data Mart (CDM) and HealthCore, Inc, which is a wholly owned, independently operated subsidiary of Elevance Health, Inc (formerly Anthem, Inc). The Medicare data was comprised of a 20% random sample of national fee-for-service data between 2007 to 2017 which included beneficiaries over the age of 65 with at least one month in which they were simultaneously enrolled in Parts A, B, and D fee-for-service coverage. The Medicaid data consisted of fee-for-service data from the five largest Medicaid plans (Florida, New York, Pennsylvania, Ohio, and California) from 1999 to 2012. The CDM data were comprised of anonymized patient-level insurance data from 2000 to 2017. The HealthCore data were comprised of beneficiaries from 2006 to 2018, including those with commercial plans from 14 states and those with Medicare plans managed by Elevance Health.

### Inclusion criteria

As outlined by Lewis et al., inclusion criteria were defined by beneficiaries with inpatient, outpatient, and prescription medication benefits, except for beneficiaries of HealthCore, for which only medical coverage was required for inclusion.^5^ Beneficiaries were also required to have their date of birth and sex recorded in the database.

### Prevalence algorithm definition

The algorithm used to compute the pooled claims database was defined by 1 IBD diagnosis by any provider regardless of specialty combined with ≥1 IBD cases diagnosed by a gastroenterologist or surgeon or a prescription for an IBD therapy in the absence of another disease indication.^9^ Only beneficiaries with a minimum of 4 years of continuous benefits were included in the prevalence estimate.

### CD and UC definition

Beneficiaries were categorized as having CD or UC if the most recent diagnosis was the same as the most frequent diagnosis on or before the date of measurement. If the number of CD diagnoses equaled that of UC diagnoses or the most recent diagnosis was not the same as the most frequent, the beneficiary was categorized as IBD, not further specified.

### Statistical analyses

The American Community Survey 1-year data from the US Census were utilized to define overall state-level populations stratified by age and sex in 2023.^10^ In order to align with the 9 age categories utilized in the pooled claims data, population counts were summed according to the following age groups: (1) “Under 5 years” and “5 to 9 years”; (2) “10 to 14 years”, “15 to 17 years”, and “18 and 19 years”; (3) “20 years”, “21 years”, “22 to 24 years”, and “25 to 29 years”; (4) “30 to 34 years” and “35 to 39 years”; (5) “40 to 44 years” and “45 to 49 years”; (6) “50 to 54 years” and “55 to 59 years”; (7) “60 and 61 years” and “62 to 64 years”; (8) “65 and 66 years”, “67 to 69 years”, “70 to 74 years”, and “75 to 79 years”; (9) “80 to 84 years” and “85 years and over”. These data were then merged with the age/sex-stratified and insurance-weighted national prevalence estimates for IBD as follows.^5^

Equation 1 was used to generate the state-specific prevalence accounting for the age, sex, and racial distribution of the state, where *r* = number of racial groups, *i* = number of age- and sex-strata, *Pi* = national prevalence of IBD in the *i^th^* stratum, *Ni* = state population in the *i^th^* stratum, *Rr* = proportion of the state population that is in the *r^th^* race group, and *Wr* is a race-specific weight applied to the national prevalence of IBD. State-specific census data were used to estimate *Rr*, the proportion of the state population that was Asian, Black, Hispanic, or White. To compute *Rr*, we used only population estimates from those people reporting a single race or ethnicity. For this analysis, it was assumed that these were the only 4 racial groups. The race-specific weights, *Wr*, were generated by dividing the national race-specific prevalence of IBD by the national overall prevalence data as reported in Lewis et al.^5^ Thus, we used the following weights: for Asian 403/721, for Black 504/721, for Hispanic 458/721, and for White 812/721. The estimated number of persons with IBD in each state was then divided by the total state population and multiplied by 100 000 to yield an age-, sex-, and race-standardized estimate of the prevalence of IBD per 100 000 population for each state and DC, which was displayed in heat maps for visualization and comparison across states.


(Equation 1) $$\frac{\left(\sum_{r=1}^{4}(\sum_{i=1}^{18}Pi*Ni)*Rr*Wr\right)}{\mathrm{Population}\;of\;\mathrm{State}}*100\;000$$

## Results

We analyzed data for 14 420 692 individuals with 4 or more years of continuous insurance, of whom 115 715 met the primary definition of IBD requiring ≥2 diagnoses or 1 diagnosis and a prescription for an IBD medication. The age-, sex- and race-weighted prevalence of IBD ranged from 4187 persons in the District of Columbia to 229 597 persons in California, with the highest absolute number of persons with IBD estimated to be in the 4 most populous states including California, Texas, Florida, and New York. The statewide absolute prevalence estimates are visually mapped in Figure 1 for IBD and Figure S1a and 1b for CD and UC, respectively, with tabular data summarized in Table S1.

**Figure 1:**
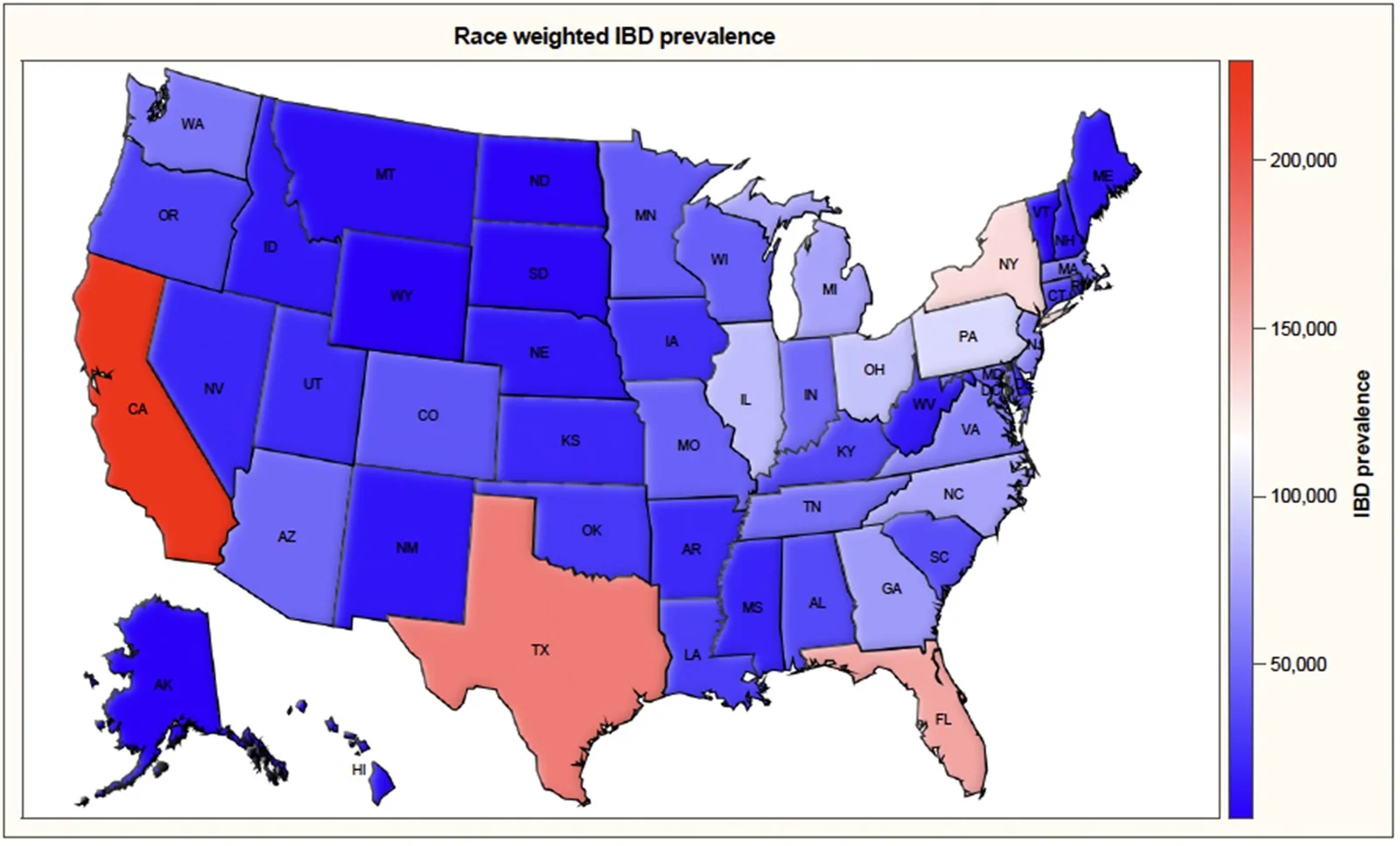
Heat map of race-weighted IBD absolute prevalence by state. IBD, inflammatory bowel disease.

IBD prevalence per 100 000 population ranged from 571 in Hawaii to 877 in Maine. The statewide estimates of prevalence per 100 000 population are summarized in Figure 2 for IBD and Figure S2a and 2b for CD and UC, respectively, with tabular data summarized in Table S1. The prevalence of IBD per 100 000 population was higher in Northern and Eastern states, including Maine, Vermont, New Hampshire, West Virginia, and Montana, whereas the prevalence of IBD per 100 000 population was lower in Southern and Western states, including Hawaii, Texas, California, District of Columbia, and New Mexico. The ratio of UC to CD was generally similar across states, ranging from 1.18 in Utah to 1.31 in Maine.

**Figure 2:**
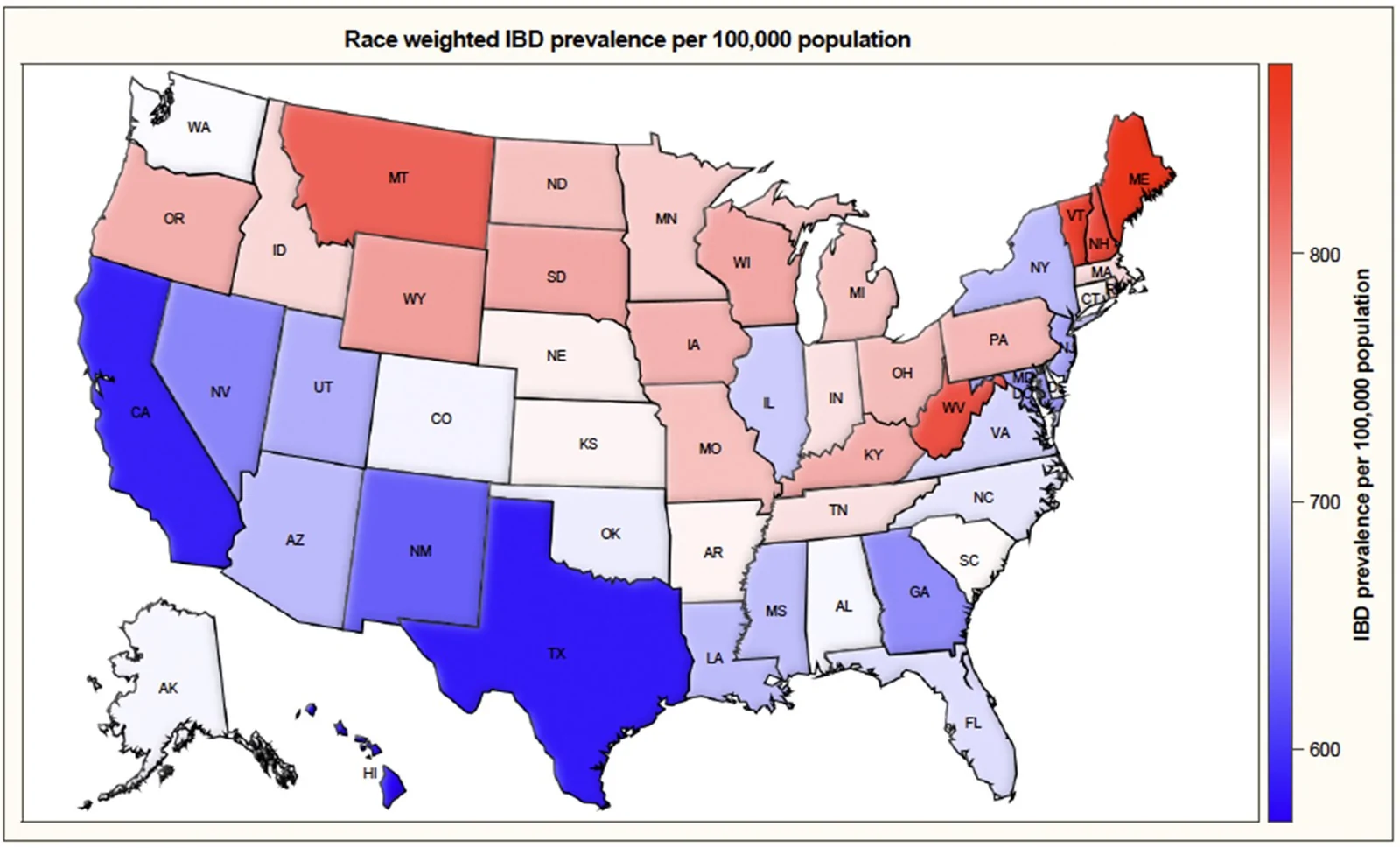
Heat map of race-weighted IBD prevalence per 100 000 population by state. IBD, inflammatory bowel disease.

## Conclusion

In this study, we leveraged prior work pooling data from commercial, Medicare, and Medicaid insurance plans to generate data on race-weighted IBD prevalence by state. We found that the highest absolute numbers of people with IBD correlated with populations by state. However, when adjusted for population size, the prevalence of IBD was highest in Northern and Eastern states. This is most evident for California and Texas which are ranked first and second for total number of residents with IBD but only 49th and 50th, respectively, for IBD prevalence per 100 000 population. Notably, the states with higher IBD prevalence per 100 000 population were observed to have a greater proportion of White residents.

Given that our national IBD prevalence estimates were approximately 4-fold higher than those reported by Upadhyay et al., the state-wide estimates were similarly multiple folds higher. Similarly, higher prevalences were reported in Northern states with Upadhyay et al. reporting highest IBD prevalence per 100 000 population in Maine and Rhode Island and lowest IBD prevalence per 100 000 population in Hawaii and New Mexico.^7^ Our estimates are consistent with those in prior studies that have reported higher incidences of IBD in women residing in northern latitudes within the United States^11^ as well as in residents of Midwestern and Eastern states.^11^

Our study has several strengths, including the use of multiple datasets representative of varying demographics and beneficiaries, which enhances the generalizability of the data. This study also captures race-weighted adjustments to better account for racial demographics in the prevalence projections. Further, our study employs a previously validated method for identifying beneficiaries with an IBD diagnosis in comparison to Upadhyay et al. who utilized an alternative IBD inclusion criterion of ≥2 nonancillary outpatient claims at least 30 days apart for IBD or 1 inpatient claim.^7^

State-level estimates of IBD prevalence provide important insights into the geographic distribution of disease burden and have implications for health care delivery and policy. Understanding the state-level distribution of individuals living with IBD can inform the allocation of gastroenterology specialists, development of specialized IBD centers, and expansion of infusion and multidisciplinary care services in regions with higher disease burden. Given the substantial health care costs associated with IBD and its therapies, state-level prevalence estimates may also support health systems, payers, and policymakers in anticipating resource needs and guiding strategic planning. Finally, these data provide important information for public health and patient advocacy efforts by quantifying the burden of disease within individual states and supporting initiatives aimed at improving access to care for individuals living with IBD.

However, this study is limited by the constraints of the merged claims dataset, which does not include uninsured individuals or those with insurance through the Department of Veterans Affairs. As discussed previously,^5^ it is unknown how the inclusion of these populations would impact the prevalence estimates. Further, the Medicaid data were limited to include data from the 5 states with the largest Medicaid plans, leading to an absence of Medicaid population representation outside of the states of Florida, New York, Pennsylvania, Ohio, and California. To the extent that age, sex, and racial distribution of Medicaid beneficiaries are similar across states, we anticipate that this should have relatively little impact on the results. Additionally, the race-weighted prevalence estimates were based on the assumption that only 4 racial groups are present and further categorized only for those study participants reporting a single race or ethnicity, an approach which may have been subject to reporting bias, misidentification, and underrepresentation of those not represented by a single racial group. It is also important to note that the CDM dataset uses a “black box” proprietary imputation algorithm to ascribe race,^12^ which may have had an impact on our estimations. Further, interpretations of data based on race classifications must be thoughtfully evaluated because race is known to be a social construct. Last, the study identified IBD prevalence on the basis of a claims-based algorithm that prioritized specificity over sensitivity. As previously reported, this algorithm may have resulted in an approximately 12% underestimation of the prevalence.^5^

In summary, the most populous states are projected to have the largest IBD populations. However, because the prevalence of IBD differs by race and the racial diversity of states vary widely, Northern and Eastern states with higher proportions of White residents are estimated to have the highest proportion of their population living with IBD.

## Supplementary Material

izag076_Supplementary_Data

## Data Availability

The data underlying this article were provided by Optum, Elevance Health, and the Center for Medicare and Medicaid Services under license and are not able to be shared by the authors.
