# Supplementary material for: Prevalence of inflammatory bowel disease across states: estimates based on claims database
Source: Inflamm Bowel Dis. 2026 May 18;32(10):2061–5. doi: 10.1093/ibd/izag076 (PMC13626250; doi:10.1093/ibd/izag076)
Supplement: Supplementary file 1 [file izag076_supplementary_data.docx]

**Supplemental Table 1.** State-specific estimated prevalence of IBD

| **State** | **State population** | **Prevalence of IBD per 100,000** | **Prevalence of CD per 100,000** | **Prevalence of UC per 100,000** | **Absolute prevalence of IBD per state** | **Absolute prevalence of CD per state** | **Absolute prevalence of UC per state** | **Rank prevalence of IBD per 100,000** | **Rank absolute prevalence of IBD** |
| --- | --- | --- | --- | --- | --- | --- | --- | --- | --- |
| CA | 38,965,193 | 589.2 | 248.9 | 309.2 | 229,597 | 96,967 | 120,471 | 49 | 1 |
| TX | 30,503,301 | 585.8 | 250.4 | 304.5 | 178,692 | 76,384 | 92,892 | 50 | 2 |
| FL | 22,610,726 | 703.5 | 289.9 | 375.8 | 159,065 | 65,553 | 84,976 | 35 | 3 |
| NY | 19,571,216 | 682.8 | 285.3 | 361.1 | 133,639 | 55,838 | 70,677 | 40 | 4 |
| PA | 12,961,683 | 768.7 | 319.1 | 408.5 | 99,639 | 41,360 | 52,951 | 12 | 5 |
| OH | 11,785,935 | 765.8 | 319.6 | 405.3 | 90,253 | 37,665 | 47,772 | 13 | 6 |
| IL | 12,549,689 | 692.5 | 290.4 | 365.2 | 86,907 | 36,446 | 45,837 | 37 | 7 |
| NC | 10,835,491 | 709.5 | 297.9 | 373.8 | 76,878 | 32,282 | 40,506 | 34 | 8 |
| MI | 10,037,261 | 761.3 | 317.2 | 403.5 | 76,415 | 31,838 | 40,498 | 16 | 9 |
| GA | 11,029,227 | 654.7 | 277.7 | 342.4 | 72,210 | 30,627 | 37,763 | 45 | 10 |
| NJ | 9,290,841 | 670.7 | 280.4 | 354.6 | 62,318 | 26,050 | 32,950 | 43 | 11 |
| VA | 8,715,698 | 697.6 | 293.2 | 367.3 | 60,797 | 25,555 | 32,012 | 36 | 12 |
| WA | 7,812,880 | 721.1 | 303.6 | 379.3 | 56,341 | 23,720 | 29,637 | 29 | 13 |
| TN | 7,126,489 | 742.9 | 312.1 | 391.3 | 52,943 | 22,240 | 27,888 | 21 | 14 |
| MA | 7,001,399 | 746.9 | 312.5 | 394.6 | 52,290 | 21,878 | 27,628 | 20 | 15 |
| IN | 6,862,199 | 742.6 | 312.2 | 390.9 | 50,959 | 21,423 | 26,825 | 22 | 16 |
| AZ | 7,431,344 | 682.5 | 285.2 | 360.9 | 50,722 | 21,195 | 26,821 | 41 | 17 |
| MO | 6,196,156 | 763.2 | 319.2 | 403.2 | 47,286 | 19,780 | 24,986 | 14 | 18 |
| WI | 5,910,955 | 778.9 | 324.4 | 413.0 | 46,042 | 19,175 | 24,410 | 8 | 19 |
| MN | 5,737,915 | 755.5 | 316.2 | 399.1 | 43,349 | 18,142 | 22,901 | 18 | 20 |
| CO | 5,877,610 | 716.8 | 303.7 | 375.3 | 42,131 | 17,850 | 22,056 | 32 | 21 |
| MD | 6,180,253 | 661.0 | 277.1 | 348.7 | 40,849 | 17,126 | 21,552 | 44 | 22 |
| SC | 5,373,555 | 726.3 | 303.0 | 384.6 | 39,029 | 16,281 | 20,665 | 27 | 23 |
| AL | 5,108,468 | 719.0 | 301.1 | 379.6 | 36,732 | 15,381 | 19,393 | 30 | 24 |
| KY | 4,526,154 | 776.7 | 325.6 | 409.7 | 35,153 | 14,737 | 18,545 | 9 | 25 |
| OR | 4,233,358 | 774.0 | 322.9 | 409.9 | 32,766 | 13,669 | 17,351 | 10 | 26 |
| LA | 4,573,749 | 683.8 | 287.3 | 360.1 | 31,277 | 13,142 | 16,472 | 39 | 27 |
| OK | 4,053,824 | 713.3 | 301.1 | 374.3 | 28,918 | 12,207 | 15,175 | 33 | 28 |
| CT | 3,617,176 | 730.5 | 304.0 | 387.5 | 26,425 | 10,996 | 14,018 | 25 | 29 |
| IA | 3,207,004 | 772.7 | 322.9 | 408.6 | 24,780 | 10,355 | 13,102 | 11 | 30 |
| UT | 3,417,734 | 675.2 | 292.9 | 346.9 | 23,076 | 10,009 | 11,856 | 42 | 31 |
| AR | 3,067,732 | 730.6 | 306.3 | 385.3 | 22,412 | 9,396 | 11,822 | 24 | 32 |
| KS | 2,940,547 | 729.9 | 307.0 | 384.1 | 21,463 | 9,027 | 11,293 | 26 | 33 |
| NV | 3,194,176 | 651.4 | 273.4 | 343.4 | 20,808 | 8,734 | 10,970 | 46 | 34 |
| MS | 2,939,690 | 684.9 | 287.7 | 360.7 | 20,134 | 8,458 | 10,604 | 38 | 35 |
| WV | 1,770,071 | 841.3 | 347.3 | 448.9 | 14,892 | 6,148 | 7,945 | 4 | 36 |
| ID | 1,964,726 | 748.9 | 315.2 | 393.9 | 14,714 | 6,194 | 7,738 | 19 | 37 |
| NE | 1,978,379 | 732.1 | 308.2 | 385.0 | 14,483 | 6,097 | 7,616 | 23 | 38 |
| NM | 2,114,371 | 628.2 | 261.9 | 332.8 | 13,282 | 5,537 | 7,036 | 47 | 39 |
| ME | 1,395,722 | 876.8 | 359.5 | 470.2 | 12,237 | 5,018 | 6,562 | 1 | 40 |
| NH | 1,402,054 | 848.4 | 350.4 | 452.6 | 11,895 | 4,913 | 6,346 | 3 | 41 |
| MT | 1,132,812 | 826.9 | 343.5 | 439.2 | 9,367 | 3,891 | 4,975 | 5 | 42 |
| RI | 1,095,962 | 759.3 | 316.4 | 402.4 | 8,322 | 3,467 | 4,410 | 17 | 43 |
| HI | 1,435,138 | 570.9 | 236.1 | 304.2 | 8,194 | 3,389 | 4,366 | 51 | 44 |
| DE | 1,031,890 | 725.9 | 300.1 | 386.9 | 7,491 | 3,097 | 3,993 | 28 | 45 |
| SD | 919,318 | 779.1 | 325.7 | 411.8 | 7,162 | 2,994 | 3,786 | 7 | 46 |
| ND | 783,926 | 762.7 | 322.1 | 400.1 | 5,979 | 2,525 | 3,136 | 15 | 47 |
| VT | 647,464 | 860.2 | 354.1 | 459.9 | 5,569 | 2,293 | 2,978 | 2 | 48 |
| AK | 733,406 | 718.5 | 306.5 | 374.3 | 5,270 | 2,248 | 2,745 | 31 | 49 |
| WY | 584,057 | 784.2 | 327.0 | 415.4 | 4,580 | 1,910 | 2,426 | 6 | 50 |
| DC | 678,972 | 616.7 | 267.0 | 317.6 | 4,187 | 1,813 | 2,156 | 48 | 51 |
